# Expression of NAD(P)H quinone dehydrogenase 1 (NQO1) is increased in the endometrium of women with endometrial cancer and women with polycystic ovary syndrome

**DOI:** 10.1111/cen.13436

**Published:** 2017-08-18

**Authors:** William Atiomo, Mohamad Nasir Shafiee, Caroline Chapman, Veronika M. Metzler, Jad Abouzeid, Ayşe Latif, Amy Chadwick, Sarah Kitson, Vanitha N. Sivalingam, Ian J. Stratford, Catrin S. Rutland, Jenny L. Persson, Niels Ødum, Pablo Fuentes‐Utrilla, Jennie N. Jeyapalan, David M. Heery, Emma J. Crosbie, Nigel P. Mongan

**Affiliations:** ^1^ Faculty of Medicine and Health Sciences Division of Obstetrics and Gynaecology and Child Health School of Medicine Queen's Medical Centre Nottingham University Hospital Nottingham UK; ^2^ Faculty of Medicine Department Obstetrics and Gynaecology UKM Medical Centre Cheras Kuala Lumpur Malaysia; ^3^ Faculty of Medicine and Health Sciences School of Veterinary Medicine and Science University of Nottingham Nottingham UK; ^4^ Faculty of Biology, Medicine and Health Division of Pharmacy and Optometry School of Health Sciences University of Manchester Manchester UK; ^5^ Faculty of Biology Division of Molecular & Clinical Cancer Sciences Medicine and Health University of Manchester Manchester UK; ^6^ Department of Obstetrics and Gynaecology Central Manchester University Hospitals NHS Foundation Trust Manchester Academic Health Science Centre Manchester UK; ^7^ Manchester School of Pharmacy University of Manchester Manchester UK; ^8^ Clinical Research Center Lund University Malmö Sweden; ^9^ Department of Molecular Bology Umeå University Umeå Sweden; ^10^ Department of Immunology and Microbiology University of Copenhagen Kobenhavn Denmark; ^11^ Edinburgh Genomics University of Edinburgh Edinburgh UK; ^12^ School of Pharmacy University of Nottingham Nottingham UK; ^13^ Department of Pharmacology Weill Cornell Medicine New York NY USA

**Keywords:** endometrial cancer, endometrium, NQO1, polycystic ovary syndrome

## Abstract

**Objective:**

Women with a prior history of polycystic ovary syndrome (PCOS) have an increased risk of endometrial cancer (EC).

**Aim:**

To investigate whether the endometrium of women with PCOS possesses gene expression changes similar to those found in EC.

**Design and Methods:**

Patients with EC, PCOS and control women unaffected by either PCOS or EC were recruited into a cross‐sectional study at the Nottingham University Hospital, UK. For RNA sequencing, representative individual endometrial biopsies were obtained from women with EC, PCOS and a woman unaffected by PCOS or EC. Expression of a subset of differentially expressed genes identified by RNA sequencing, including NAD(P)H quinone dehydrogenase 1 (*NQO1*), was validated by quantitative reverse transcriptase PCR validation (n = 76) and in the cancer genome atlas UCEC (uterine corpus endometrioid carcinoma) RNA sequencing data set (n = 381). The expression of NQO1 was validated by immunohistochemistry in EC samples from a separate cohort (n = 91) comprised of consecutive patients who underwent hysterectomy at St Mary's Hospital, Manchester, between 2011 and 2013. A further 6 postmenopausal women with histologically normal endometrium who underwent hysterectomy for genital prolapse were also included. Informed consent and local ethics approval were obtained for the study.

**Results:**

We show for the first that *NQO1* expression is significantly increased in the endometrium of women with PCOS and EC. Immunohistochemistry confirms significantly increased NQO1 protein expression in EC relative to nonmalignant endometrial tissue (*P *< .0001).

**Conclusions:**

The results obtained here support a previously unrecognized molecular link between PCOS and EC involving NQO1.

## 1 INTRODUCTION

Endometrial cancer (EC) is the most common gynaecological cancer affecting women in the United States, with an estimated 60 050 new cases in 2016.^1^ The incidence of EC has increased by over 65% since the late 1970s correlating with rising incidence of obesity and increased longevity.^2^, ^3^ EC is usually treated by hysterectomy, but surgery carries increased risk in obese women and renders premenopausal women infertile. In addition to its negative impact on quality of life, EC poses a significant economic burden on health services.

Polycystic ovary syndrome (PCOS) is the commonest female endocrinopathy affecting 3%‐20% of women of reproductive age.^4^, ^5^ Women with PCOS experience obesity, infrequent menstrual periods, infertility, excess systemic androgens, insulin resistance and hirsutism, and have enlarged ovaries with multiple small follicles on ultrasound imaging.^6^ Women with PCOS have an increased risk of type 2 diabetes in later life and a threefold to fourfold increased risk of EC.^7^, ^8^

The exact mechanisms that predispose PCOS women to EC remain unknown. Current hypotheses include a link between obesity and elevated oestrogen levels, inflammation, type 2 diabetes and hyperinsulinaemia.^9^ In recently published studies, we found altered expression of genes involved in insulin signalling (IGF‐1, IGFBP1 and PTEN) and lipogenic gene regulation in the endometrium and serum of women with PCOS and EC compared with controls.^10^, ^11^ Genes related to immunoregulation/inflammation,^12^ antioxidants^9^ and impaired progesterone‐mediated decidualization^13^ have also been suggested as possible mechanisms linking PCOS and EC.

This complexity highlights the need to characterize the transcriptome of the endometrium of women with PCOS to advance understanding of mechanisms linking PCOS and EC. To our knowledge, comparative transcriptomic, proteomic or metabolomic studies of patients with PCOS and EC are as yet unavailable. The aim of this proof of principle study was to perform comparative RNA sequencing profiling of endometrial biopsies from women with PCOS and EC and validate these findings in a large cohort of patients with EC.

## 2 METHODS

### 2.1 Study design, patient recruitment, sample acquisition and processing

Details of the methods used in patient recruitment for the Nottingham cohort have been previously described in detail elsewhere.^10^, ^11^ Briefly, patients (N = 76) were recruited into to a cross‐sectional study conducted within the division of Obstetrics and Gynaecology and Child Health, at Nottingham University Hospital in the United Kingdom. Participants were prospectively recruited from July 2013 to February 2014. Research ethics approval was obtained from the National Research Ethics Service, East Midlands‐Northampton committee (13/EM/0119) prior to commencement of recruitment. The project was also reviewed and approved by the relevant local ethics committees at the University of Nottingham. The Helsinki Declaration was strictly observed. Informed consent was obtained from all participants. For RNAseq, representative endometrial biopsies were obtained from individual women from each arm, specifically with EC (age 43, BMI = 35.9), polycystic ovary syndrome (age = 41, BMI = 35.9) and an age‐ and BMI‐matched women unaffected by PCOS or EC (age = 42, BMI 32.01) and stored immediately in RNAlater (Sigma‐Aldrich, Gillingham, UK). For qRT‐PCR validation, participants were recruited in three arms: PCOS (n = 26), EC (n = 25) and control (n = 25). The participants were between 19 and 84 years of age and not on any hormonal treatment. Pregnancy was excluded prior to the recruitment using standard urine pregnancy tests. The EC group consisted of women with histopathologically proven endometrioid (type 1) adenocarcinoma of the endometrium undergoing total hysterectomy (by laparotomy or laparoscopically) who had not received previous neo‐adjuvant chemo‐ or radiotherapy. Women were excluded from the study for prior history of papillary serous adenocarcinoma or metachronous cancers of the ovary, endometrium or cervix. The PCOS cohort was defined using the Rotterdam European Society for Human Reproduction and Embryology (ESHRE) and the American Society of Reproductive Medicine (ASRM) criteria. Baseline demographic details, blood pressure, weight and body mass index were calculated (kg/m^2^), the Ferriman Gallwey hirsuitism score was recorded, and hip‐waist circumference was recorded (cm) with participants wearing indoor clothing. The control group comprised of healthy women without EC or PCOS, not on any hormonal therapy, undergoing pelvic surgery for benign indications. The following clinical parameters were measured in participants using standard UK National Health Service services: fasting blood glucose, low‐density lipoprotein, high‐density lipoprotein, triglycerides, sex hormone bind globulin, testosterone, follicle stimulating hormone, luteinizing hormone, prolactin, 17‐hydroxy‐progesterone and thyroid function. A Pipelle^®^ endometrial catheter was used to biopsy endometrial tissue, and samples were snap frozen at −80 C for subsequent processing. Patient characteristics and the results of endocrine and metabolic assays were as previously reported (Table 1).

**Table 1:** Participants' characteristic, biochemical and hormonal data of the Nottingham cohort. One‐way ANOVA test was used to determine the difference between the groups

	Control (n = 25)	PCOS (n = 26)	Endometrial cancer (n = 25)	*P* value
Age (years);Mean (SD)	45.96 (13.34)	31.88 (5.975)	62.64 (11.10)	<.0001^a^
BMI (Kg/m^2^);Mean (SD)	29.27 (2.467)	29.60 (3.116)	33.12 (5.959)	.0021^a^
WHC ratio; Mean (SD)	88.56 (3.241)	88.5 (3.992)	96.86 (12.99)	.0003^a^
Systolic BP(mm Hg);Mean (SD)	135.9 (8.729)	134.1 (7.591)	148.2 (11.42)	<.0001^a^
Diastolic BP (mm Hg);Mean (SD)	81.88 (7.563)	83.35 (7.746)	84.80 (7.455)	.4012
Fasting insulin; Mean (SD)	13.47 (6.002)	20.04 (31.79)	18.3 (16.26)	.5199
Fasting glucose; Mean (SD)	4.844 (0.4788)	5.142 (0.8363)	6.4 (1.649)	<.0001^a^
HOMA‐IR; Mean (SD)	0.1809 (0.1065)	0.2617 (0.4405)	0.3139 (0.3766)	.3871
LDL; Mean (SD)	2.820 (0.8539)	2.738 (0.8174)	2.632 (0.9919)	.7561
HDL; Mean (SD)	1.5 (0.3136)	1.415 (0.2664)	1.648 (0.3754)	.0381^a^
TG; Mean (SD)	1.344 (0.6752)	1.373 (0.4609)	1.54 (0.5694)	.4305
Total cholesterol; Mean (SD)	5.004 (0.9654)	1.373 (0.4609)	1.54 (0.5694)	.5293
FSH; Mean (SD)	17.88 (24.81)	5.008 (2.788)	49.01 (20.83)	<.0001^a^
LH; Mean (SD)	12.95 (12.04)	12.31 (11.08)	28.64 (13.08)	<.0001^a^
Testosterone; Mean (SD)	1.516 (0.6309)	2.846 (0.7089)	1.492 (0.8684)	<.0001^a^
Oestradiol; Mean (SD)	414.5 (655.8)	331.9 (261.3)	96.72 (55.39)	.0204^a^
Progesterone; Mean (SD)	6.448 (15.37)	11.2 (14.54)	1.28 (0.5292)	.0192^a^
SHBG; Mean (SD)	55.36 (38.65)	34.46 (14.03)	50.6 (17.01)	.0120^a^

a *P* value <.5 is significant are indicated.

### 2.2 RNA sequencing (RNAseq) and quantitative reverse transcriptase PCR (qRT‐PCR) analysis of patient endometrial samples

Total RNA was isolated using an RNeasy extraction kit, with on‐column DNAse digestion (Qiagen, Manchester, UK). RNA quality (RIN > 7) was confirmed using an Agilent bioanalyser. Samples were submitted to Edinburgh Genomics for library preparation and analysed using an Illumina HiSeq platform using standard protocols. For quantitative reverse transcriptase PCR validation of RNAseq results, the samples were a subset (Table 1) of the patients described previously.^10^ The expression of identified differentially expressed genes was examined in the cancer genome atlas UCEC (uterine corpus endometrioid carcinoma) RNA sequencing data set.^14^

Paired end raw reads (fastq format) were quality‐ and adapter‐filtered using the Trim‐galore wrapper for FastQC and cutadapt (http://www.bioinformatics.babraham.ac.uk/projects/trim_galore/). The retained paired reads were aligned to the Ensembl annotated HG19 human Illumina iGenome build using Tophat2, and differential gene expression was calculated for PCOS and EC specimens relative to the control specimen using Cuffdiff^15^ on the basis of fold changes >1.5 and *P*‐value <.05. Statistically significantly enriched gene ontologies and pathways for differentially expressed genes were obtained using WebGestalt and the Cytoscape Genemania plugin.^16^ Next‐generation RNAseq and associated clinical information was obtained from the cancer genome atlas endometrium cancer (UCEC) data set.^14^ Normalized RSEM expression counts scaled to library size from each patient were compiled and correlated with specific clinical features including tumour and nontumour endometrial tissue and grade were analysed using EdgeR^17^ or the Wilcoxon test with Benjamini‐Hochberg false discovery rate correction for multiple testing.

We used qRT‐PCR to validate differential endometrial gene expression identified by RNAseq in a subset of Nottingham cohort of PCOS, EC and control patients for whom mRNA and cDNA were available as previously described.^10^ The hydrolysis probe PCR reagents employed were as follows: β*‐actin*: Hs01060665_g1; *NQO1*: Hs02512143_s1. Each sample was analysed in triplicate using the Plaffl method. For qPCR experiments, unpaired *t* tests were used to compare expression between control, PCOS and EC specimens.

### 2.3 Immunohistochemistry

Samples from a separate cohort at the University of Manchester investigating prognostic biomarkers in EC using immunohistochemistry provided an opportunity to further investigate and validate the role of differentially expressed genes in EC. The Manchester cohort consisted of consecutive patients (n = 91) who underwent hysterectomy for EC at St Mary's Hospital in Manchester between 2011 and 2013, and who provided written, informed consent for their tumour samples to be stored in the BRC Biobank and used for future research. A further 6 postmenopausal women with histologically normal endometrium who underwent hysterectomy for genital prolapse were also included. The study received ethical approval from NRES Committee London ‐ Fulham (REC reference 12/LO/0364) and R&D approval (R01960) from Central Manchester University Hospitals NHS Foundation Trust. The EC cohort comprised different histological subtypes, grades and stages of EC that were fully annotated with respect to patient demographics and clinical follow‐up data. The average follow‐up for the Manchester cohort was 34 months (range 1‐64), during which time there were 19 recurrences and 23 deaths, of which 13 were EC‐specific.

Formalin‐fixed, paraffin‐embedded tissue samples were cut into 4‐μm sections for IHC analysis. This was performed using a fully automated IHC platform, Leica BOND‐MAX together with Bond™ Polymer Refine Detection kit (DS9800) and on‐board retrieval system. The sections were labelled with NQO1 (Sigma, 1:75 dilution) primary antibody according to standard validated Protocol *F* written by Leica. The detection kit was a biotin‐free, polymeric horseradish peroxidase (HRP)‐linker antibody conjugate system that detects tissue‐bound IgG primary antibodies using the chromogen 3,3′‐diaminobenzidine tetrahydrochloride hydrate (DAB) via a brown precipitate. Tissue sections were then counterstained with haematoxylin. Immunohistochemical evaluation was performed blindly by two independent observers (AL and AC) and discordant cases settled by review. NQO‐1 staining was scored using the product of the area score (proportion of positively staining tumour cells) and the intensity of staining (0‐3, 0 = zero staining, 3 = high‐intensity staining). The score range was 0‐300, and tumours were then dichotomised into low expression (score < 200) and high expression (score > 200).

### 2.4 Statistical analysis

NQO1 protein expression in normal and malignant endometrium was compared using the Mann‐Whitney *U* test. The association between NQO1 protein expression and clinical‐pathological variables in women with endometrial cancer was tested using the Mann‐Whitney *U* test for nonparametric variables and Spearman rank correlation for continuous and ordinal variables. Kaplan‐Meier curves were constructed to estimate the effect of NQO1 expression on overall, cancer‐specific and recurrence‐free survival, with curves compared using the log‐rank test. Overall survival was defined as the time between date of surgery and death from any cause, while cancer‐specific survival referred to the time interval between surgery and death from endometrial cancer. Recurrence‐free survival was defined as the time between date of surgery and first documented local or distant recurrence. Data without events were censored at date of last clinical follow‐up visit. A Cox proportional hazard regression model was used in a univariate analysis of cancer‐specific and recurrence‐free survival, after confirming that the data were complied with the proportional hazards assumption using log‐log curves. All clinical‐pathological variables with known prognostic value in endometrial cancer were included in the univariate analysis alongside NQO1.

## 3 RESULTS

### 3.1 Patient demographics for samples used for the RNA sequencing and PCR validation study

**Figure 1:**
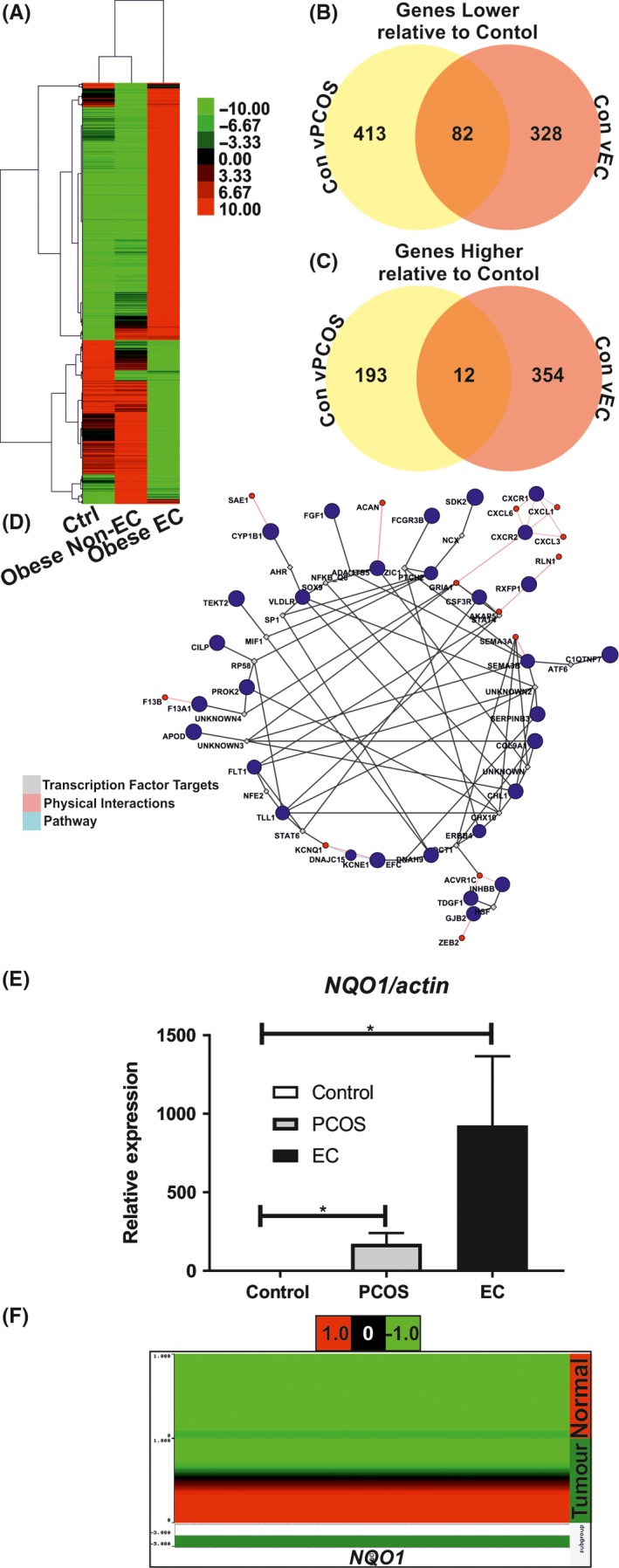
Next‐generation RNA sequencing was used to compare the transcriptome of endometrial samples from unaffected, PCOS and EC patients. Unsupervised hierarchical clustering indicates nonmalignant endometrial specimens from unaffected control and PCOS patients are most similar. Elevated gene expression is indicated in red, and lower gene expression is indicated in green (A). A subset of 94 genes comprised of 82 down‐regulated genes and 12 upregulated (B, C) are commonly dysregulated in PCOS and malignant endometrium. The Genemania cytoscape plugin was used to identify common pathways and infer potential transcriptional regulators of the gene network (D). We used qRT‐PCR (E) and the cancer genome atlas (F) to compare expression of *NQO1* normalized to actin in endometrial biopsies from PCOS (n = 25) and EC (n = 25) women relative to unaffected women (n = 25). **** = *P* <.005 by Kruskal‐Wallis nonparametric analysis of variance with Dunn's post hoc multiple comparisons test. We also analysed expression of *NQO1,* and it downstream target *p53* in the cancer genome atlas UCEC data set (N = 370 EC, N = 11 nonmalignant endometrium). Expression of *NQO1* is significantly elevated in tumour, relative to nonmalignant endometrial tissue as determined by Wilcoxon test with Benjamini‐Hochberg false discovery rate correction for multiple testing. [Colour figure can be viewed at wileyonlinelibrary.com]

Samples from three women were submitted for RNA sequencing. One patient with EC (BMI = 35.9, age 43), one PCOS patient without EC (BMI = 35.9, age 41) and an unaffected control woman (BMI = 32, age 42) were used for the RNA sequencing experiments. RNAseq identified differentially expressed genes (using standard criteria of fold changes [FC] >1.5, *P*‐value <.05) in PCOS (700 genes) and EC (776 genes) endometrial specimens relative to control nonmalignant endometrium (Figure 1A‐D, Tables S1, S2). We found that the global transcriptional profile of endometrial tissue from the woman with PCOS was most similar to the control obese woman (Figure 1). Of these genes, 94 genes were differentially expressed in both EC and PCOS relative to control endometrium (Table S1). Specifically, 12 genes were higher and 82 were lower in PCOS and EC specimens relative to control endometrium (Table S1, S2).

In the qRT‐PCR validation cohort, the BMIs of women with EC (33.12 ± 5.959 kg/m^2^), PCOS (31.88 ± 5.975 kg/m^2^) and controls (29.27 ± 2.467 kg/m^2^) were not significantly different. PCOS women were however younger (31.88 ± 5.975 years) than women with EC (62.64 ± 11.10 years) and controls (45.96 ± 13.34 years). Women with PCOS were recruited during their proliferative menstrual phase (based on their menstrual histories). We next used qRT‐PCR to validate the expression of a subset of these genes in our patient cohorts (Figure 1E, Figure S1B). We confirmed expression of *NQO1* (Figure 1E), the NQO1 target *p53* and another exemplar gene identified by RNAseq, *GJB2,* (Figure S1) was significantly increased (*P *< .05) in endometrial specimens from women with PCOS (n = 25) and EC (n = 25) as compared to control, unaffected women (n = 25). We next examined expression of these 94 genes in patients with EC using the cancer genome atlas.^17^ Of these 94 genes, 14 genes (*NQO1, SLPI, GJB2, DNAJC15, S100A8, PLEKHS1, ESPN, RSPH1, KRT5, FOXJ1, IFI27, IFI6, LGR5 and MUC16*) were significantly altered in tumour as compared to nontumour endometrial specimens (Figure S1). Expression of *NQO1* and its target *p53* mRNA are all significantly higher in primary endometrial tumour (n = 370) than nontumour (n = 11) specimens (Figure S1) as reported in the cancer genome atlas UCEC data set. The Genemania Cytoscape plugin was used to identify pathways and infer potential transcriptional regulators of the genes identified common to PCOS and EC (Figure 1D). Enriched gene ontologies defined by these differential genes were identified (Table S2). Interestingly, the significantly enriched gene ontologies included gene networks involved in microtubule motor activity and cilia function.

### 3.2 Immunohistochemistry validating the role of NQO1 in EC

**Figure 2:**
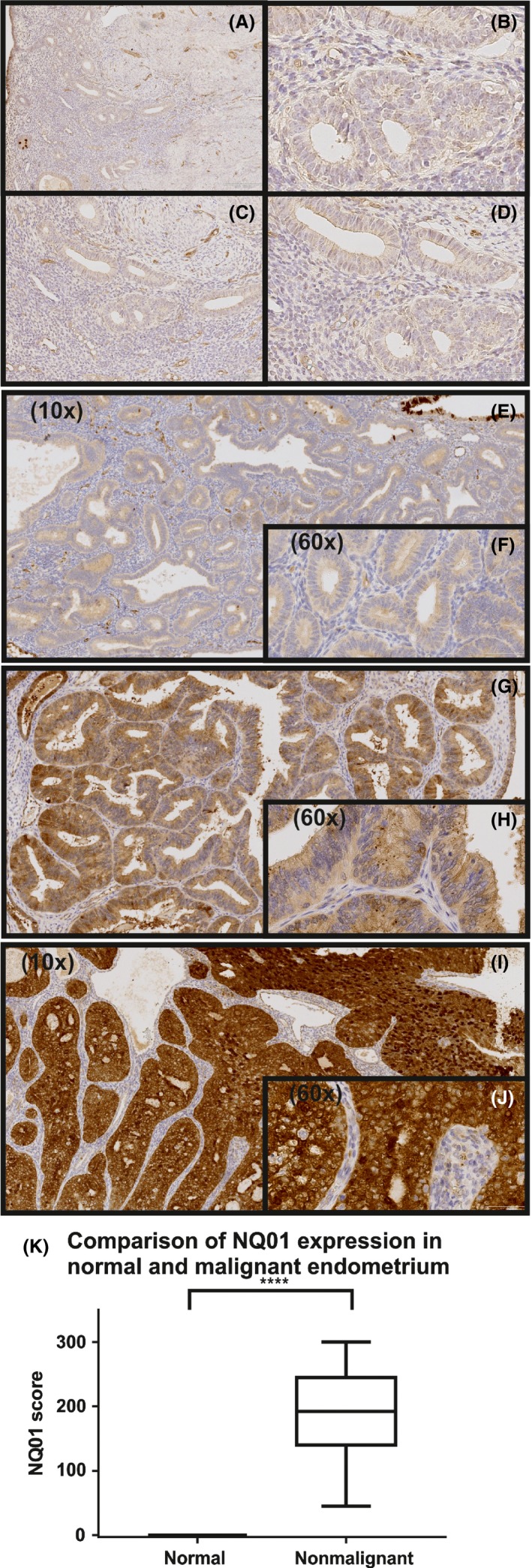
Immunohistochemistry was used to evaluate expression of NQO1 protein in nontumour (A‐D) and endometrial cancer specimens (E‐J). Representative NQO1 staining of endometrial cancer specimens with intensity scores of 1 (E, F), 2 (G, H) and 3 (I, J) at 10× and 60× magnification is shown. (F) Comparison of NQ01 expression in normal and malignant endometrium as determined by immunohistochemistry. NQ01 was expressed solely in endometrial cancers and not demonstrated in normal endometrium from control patients (*P *< .0001). [Colour figure can be viewed at wileyonlinelibrary.com]

The Manchester EC patient demographics and clinicopathological features are shown (Table 2). The control women were postmenopausal with histologically normal endometrium and underwent hysterectomy for genital prolapse. Histologically normal postmenopausal endometrium did not express NQO1 (Figure 2). In EC, there was a statistically significant association between high NQO1 expression and advancing age (Table S3). There was no statistically significant correlation between NQO1 expression and standard clinicopathological features with established prognostic value, including histological subtype, grade or stage of disease, deep myometrial invasion or LVSI (Table S3). Both type 1 (endometrioid) and type 2 (nonendometrioid) EC expressed NQO1, and there was a trend towards poorer outcomes with higher NQO1 staining; however, NQO1 expression was not associated with recurrence‐free, EC‐specific or overall survival in the Manchester cohort (Table S3, Figure S2). This may reflect the good overall prognosis of EC and an insufficient number of events to demonstrate significance.

**Table 2:** Relationship between known prognostic variables and NQO1 expression in the Manchester cohort

Characteristic	NQO1			*P* value
	All n = 91	NQO1 score <200 n = 53	NQO1 score ≥200 n = 38	
Median age at diagnosis years (IQR)	68 (58‐74)	67 (56‐72)	72.5 (63.3‐77.8)	.007^a^
Median BMI at diagnosis kg/m^2^ (IQR)	30.1 (26.1‐37.1)	30.1 (26.0‐39.1)	29.6 (26.3‐35.2)	.622
Diabetic, n (%)				
No	71 (78.0)	43 (81.1)	28 (73.7)	.629
Yes	20 (22.0)	10 (18.9)	10 (26.3)	
Histological grade, n (%)				
1	23 (25.3)	15 (28.3)	8 (21.1)	.164
2	20 (22.0)	12 (22.6)	8 (21.1)	
3	48 (52.7)	26 (49.1)	22 (57.9)	
FIGO (2009) stage, n (%)				
1	59 (64.8)	34 (64.2)	25 (65.8)	.115
2	12 (13.2)	9 (17.0)	3 (7.9)	
3	18 (19.8)	9 (17.0)	9 (23.7)	
4	2 (2.2)	1 (1.9)	1 (2.6)	
Histological type, n (%)				
Endometrioid	48 (52.7)	31 (58.4)	17 (44.7)	.100
Nonendometrioid	43 (47.3)	22 (41.5)	21 (55.3)	
Lymphovascular space invasion, n (%)				
Absent	50 (53.8)	32 (60.4)	18 (47.4)	.356
Present	38 (41.8)	18 (34.0)	20 (52.6)	
Missing data	3 (3.3)	3 (5.7)	0 (0)	
Depth of myometrial invasion, n (%)				
<50%	51 (56.0)	31 (58.5)	20 (52.6)	.412
≥ 50%	40 (44.0)	22 (41.5)	18 (47.4)	
Any adjuvant treatment, n (%)				
No	37 (40.7)	23 (43.4)	14 (36.8)	.366
Yes	54 (59.3)	30 (56.6)	24 (63.2)	

a indicates *p* values <0.01.

## 4 DISCUSSION

Known risk factors for EC include increasing age, polycystic ovary syndrome (PCOS), obesity and type 2 diabetes.^18^, ^19^, ^20^, ^21^ We and others have identified altered SREBP1,^11^ and insulin signalling in endometrial specimens from women with PCOS or EC.^10^, ^22^ Anovulatory menstrual cycles, commonly found in PCOS women, have also been linked with EC.^23^ The mechanisms are thought to involve a state of progesterone deficiency. Progesterone protects the endometrium from the mitogenic effects of oestrogen and withdrawal of progesterone triggers endometrial sloughing (menstruation), which allows the natural shedding of abnormal endometrial cells. A common systemic pathway such as an aberrant insulin signalling pathway may cause oligo/amenorrhoea as well as endometrial hyperplasia and EC independent of body mass index (BMI). In such a scenario, aberrant systemic signalling programs pro‐oncogenic transcriptional networks in the endometrium of women with PCOS that may predispose to EC. Indeed, while previous gene expression studies have investigated PCOS^24^ and EC,^25^ the exact mechanisms that predispose obese women to EC are unclear. Thus, while the epidemiological evidence supporting an association between PCOS and an increased risk of endometrial carcinogenesis is robust,^10^, ^19^, ^20^, ^21^ a definitive mechanistic link between the conditions has yet to be identified. For this reason, the goal of this study was to compare endometrial gene expression profiles from women with endometrial cancer and PCOS. The current study is the first to compare global gene expression in endometrial specimens from women with PCOS and EC. Our findings have identified 94 genes, including *NQO1*, commonly altered in endometrial specimens from women with PCOS and EC, suggesting a potential common mechanism in the disorders.

*NQO1* has an established role in the endometrium.^26^ *NQO1* encodes NAD(P)H:quinone oxidoreductase 1 in detoxification pathways^27^, ^28^, ^29^ and has been reported to activate specific quinone‐derived pharmaceuticals including mitomycin C and apaziquone.^30^, ^31^ NQO1 also acts to protect the p53 tumour suppressor protein, and many other proteins involved in proliferation from proteasomal degradation.^32^ Interestingly, missense variants in *NQO1* are implicated in many cancer types^33^, ^34^, ^35^ and more recently, increased NQO1 expression is associated with poor prognosis in ovarian^36^ and lung^37^ cancers. There is also considerable interest in NQO1 as a cancer therapeutic target. Its ability to activate cytotoxic therapies selectively within malignant tissue may be an attractive therapeutic approach.^38^ Consistent with this NQO1 null mice are more sensitive to chemical induced carcinogenesis^32^ and NQO1 plays an essential role in oncogene‐induced senescence. However, the association of overexpression of NQO1 with poorer outcomes in certain cancer types^36^, ^37^ suggests that tumours can bypass the antiproliferative actions of NQO1, potentially through the activation of pro‐carcinogenic compounds.^39^

In this current study, expression of *NQO1* was significantly higher in tumour as compared to matched nontumour specimens (Figures 1, 2). NQO1 expression is regulated by the oestrogen receptor‐α (ERα/NR3A1) and oestrogen receptor‐β (ERβ/NR3A2)^40^ and by progesterone.^41^ As outlined above, aberrant oestrogen and progesterone signalling contributes to EC risk. Related to this, the antibreast cancer selective oestrogen receptor modulator, tamoxifen, is known to increase EC risk by inducing oestrogen‐regulated gene expression^42^ and altering oestrogen metabolism in endometrial cells.^43^ NQO1 may therefore play a key role in the oestrogen‐related links between EC and PCOS.

**Figure 3:**
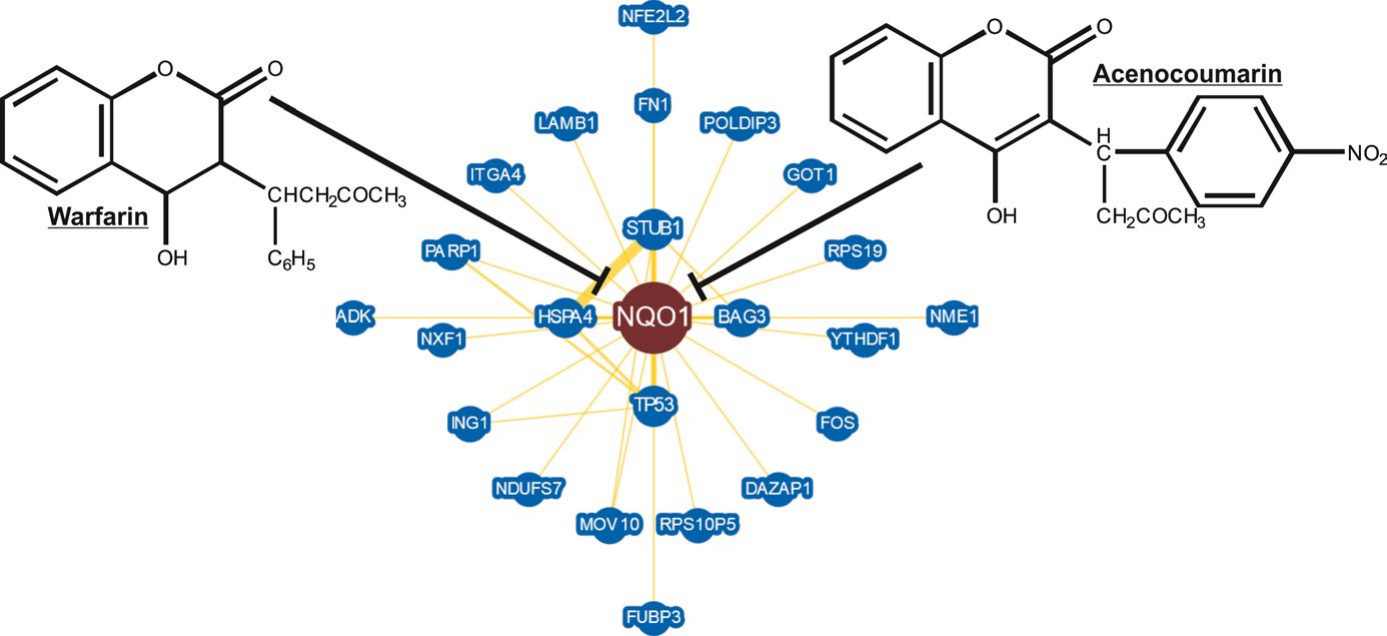
The BioGRID database^45^ reports NQO1 functionally interacts with key regulators of cell proliferation including TP53. Furthermore, the enzymatic activity of NQO1 can be inhibited by the anticoagulants, warfarin and acenocoumarin and more specific next‐generation NQO1‐inhibitors are now available. This may point to potential chemo‐preventative approaches to pharmacologically target elevated expression of NQO1 function in women with PCOS. [Colour figure can be viewed at wileyonlinelibrary.com]

In conclusion, we have used a preliminary RNAseq analysis to identify aberrant gene expression in EC and endometrium from women with PCOS. One limitation to our study was that only individual patient samples were sequenced. However, we confirmed expression of a key gene identified by RNAseq, *NQO1,* in larger cohorts of patients with PCOS and EC at the mRNA and protein levels. In this study, increased NQO1 expression was not associated with standard prognostic clinicopathological features including tumour type, grade, stage and myometrial invasion lymphovascular space invasion (LVSI). Indeed, NQO1 was expressed ubiquitously in most tumours and was not associated with recurrence‐free, endometrial cancer‐specific or overall survival. However, *NQO1* expression, like that of 93 other transcripts, was deregulated in both PCOS and EC. This supports the hypothesis that PCOS can induce gene expression changes in the endometrium that resembles EC. It is possible such changes in gene expression contribute to the increased risk of EC in women with PCOS. NQO1 represents a potential therapeutic target in EC. NQO1 is inhibited by dicoumarol, and more specific next‐generation NQO1‐inhibitors are now available (Figure 3).^44^ Therefore, the preclinical testing of such compounds for the treatment of EC and prevention of future EC in PCOS is warranted. Finally, further studies are now warranted to examine NQO1 protein expression in endometrial biopsies from women with PCOS in a prospective study to determine whether NQO1 may be useful to distinguish women at increased risk of developing EC.

## CONFLICT OF INTERESTS

The authors confirm no conflict of interests related to this study.

## AUTHOR CONTRIBUTIONS

WA, EJC, MNS and NPM involved in study design, patient recruitment and project management. MNS, CC, VMM, JA, AL, AC, SK, VS, IS, CSR, JLP, NO, PF‐U, DMH, CSR, NPM, EJC and WA conducted the experiments and data analysis. WA, EJC, JA, VNS, AL, NO, JLP, JJ, CSR, IJS, DMH and NPM wrote the manuscript.
