# Supplementary material for: Expression of NAD(P)H quinone dehydrogenase 1 (NQO1) is increased in the endometrium of women with endometrial cancer and women with polycystic ovary syndrome
Source: Clin Endocrinol (Oxf). 2017 Aug 18;87(5):557–65. doi: 10.1111/cen.13436 (PMC5697576; doi:10.1111/cen.13436)
Supplement: Supplementary file 3 [file CEN-87-557-s003.docx]

**Supplemental Table 1. Common differentially expressed genes in PCOS and EC relative to control endometrium**

|  |  | **Fold Change (log2)** | |
| --- | --- | --- | --- |
| **Symbol** | **Gene** | **Con v PCOS** | **Con v EC1** |
| *NQO1* | NAD(P)H dehydrogenase, quinone 1 | 1 | 2 |
| *NCEH1* | neutral cholesterol ester hydrolase 1 | 1 | 2 |
| *VLDLR* | very low density lipoprotein receptor | 1 | 2 |
| *PLVAP* | plasmalemma vesicle associated protein | 1 | 2 |
| *FLT1* | fms-related tyrosine kinase 1 | 1 | 3 |
| *GJB2* | gap junction protein, beta 2, 26kDa | 1 | 3 |
| *VNN1* | vanin 1 | 2 | 7 |
| *DNAJC15* | DnaJ (Hsp40) homolog, subfamily C, member 15 | 2 | 2 |
| *TLL1* | tolloid-like 1 | 3 | 5 |
| *VWDE* | von Willebrand factor D and EGF domains | 3 | 4 |
| *TDGF1* | teratocarcinoma-derived growth factor 1 | 3 | 5 |
| *HLA-DQA1* | major histocompatibility complex, class II, DQ alpha 1 | 4 | 6 |
| *SNHG5* | small nucleolar RNA host gene 5 | -3 | -2 |
| *DYDC2* | DPY30 domain containing 2 | -2 | -5 |
| *INHBB* | inhibin, beta B | -2 | -5 |
| *PKHD1L1* | polycystic kidney and hepatic disease 1 (autosomal recessive)-like 1 | -2 | -7 |
| *IGF2* | insulin-like growth factor 2 | -2 | -3 |
| *CFD* | complement factor D (adipsin) | -2 | -5 |
| *ESPN* | espin | -2 | -4 |
| *RSPH1* | radial spoke head 1 homolog (Chlamydomonas) | -2 | -3 |
| *H19* | H19, imprinted maternally expressed transcript (non-protein coding) | -2 | -4 |
| *COL9A1* | collagen, type IX, alpha 1 | -2 | -6 |
| *FAM179A* | family with sequence similarity 179, member A | -2 | -5 |
| *ADAMTS5* | ADAM metallopeptidase with thrombospondin type 1 motif, 5 | -2 | -3 |
| *ERBB4* | erb-b2 receptor tyrosine kinase 4 | -2 | -3 |
| *SLPI* | secretory leukocyte peptidase inhibitor | -2 | -3 |
| *KCNE1* | potassium channel, voltage gated subfamily E regulatory beta subunit 1 | -2 | -7 |
| *DNAH12* | dynein, axonemal, heavy chain 12 | -2 | -7 |
| *IQUB* | IQ motif and ubiquitin domain containing | -2 | -5 |
| *DNAH2* | dynein, axonemal, heavy chain 2 | -2 | -5 |
| *WDR16* | CFAP52 cilia and flagella associated protein 52 | -2 | -6 |
| *FHAD1* | forkhead-associated (FHA) phosphopeptide binding domain 1 | -2 | -6 |
| *FGF1* | fibroblast growth factor 1 (acidic) | -2 | -2 |
| *CCDC19* | CFAP45 cilia and flagella associated protein 45 | -2 | -4 |
| *KIF19* | kinesin family member 19 | -2 | -4 |
| *ENKUR* | enkurin, TRPC channel interacting protein | -2 | -6 |
| *KRT5* | keratin 5, type II | -2 | -8 |
| *TMEM232* | transmembrane protein 232 | -2 | -5 |
| *C1QTNF7* | C1q and tumor necrosis factor related protein 7 | -2 | -3 |
| *CILP* | cartilage intermediate layer protein, nucleotide pyrophosphohydrolase | -2 | -8 |
| *WDR96* | CFAP43 cilia and flagella associated protein 43 | -2 | -5 |
| *ZMYND10* | zinc finger, MYND-type containing 10 | -2 | -5 |
| *FAM92B* | family with sequence similarity 92, member B | -2 | -6 |
| *USP32P2* | ubiquitin specific peptidase 32 pseudogene 2 | -2 | -4 |
| *RSPH4A* | radial spoke head 4 homolog A (Chlamydomonas) | -1 | -6 |
| *DUOX1* | dual oxidase 1 | -1 | -6 |
| *C6orf165* | cilia and flagella associated protein 206 | -1 | -5 |
| *CCDC108* | coiled-coil domain containing 108 | -1 | -5 |
| *KNDC1* | kinase non-catalytic C-lobe domain (KIND) containing 1 | -1 | -4 |
| *DZIP1L* | DAZ interacting zinc finger protein 1-like | -1 | -4 |
| *TEKT2* | tektin 2 (testicular) | -1 | -4 |
| *APOD* | apolipoprotein D | -1 | -5 |
| *FOXJ1* | forkhead box J1 | -1 | -2 |
| *CCDC17* | coiled-coil domain containing 17 | -1 | -5 |
| *CYP1B1* | cytochrome P450, family 1, subfamily B, polypeptide 1 | -1 | -6 |
| *DNAH10* | dynein, axonemal, heavy chain 10 | -1 | -5 |
| *BAIAP2* | BAI1-associated protein 2 | -1 | -3 |
| *F13A1* | coagulation factor XIII, A1 polypeptide | -1 | -5 |
| *AGR3* | anterior gradient 3, protein disulphide isomerase family member | -1 | -4 |
| *PNMAL1* | paraneoplastic Ma antigen family-like 1 | -1 | -6 |
| *IFI27* | interferon, alpha-inducible protein 27 | -1 | -4 |
| *PTCH2* | patched 2 | -1 | -4 |
| *VGLL3* | vestigial-like family member 3 | -1 | -3 |
| *CHL1* | cell adhesion molecule L1-like | -1 | -5 |
| *RXFP1* | relaxin/insulin-like family peptide receptor 1 | -1 | -3 |
| *PCAT19* | prostate cancer associated transcript 19 (non-protein coding) | -1 | -5 |
| *IFI6* | interferon, alpha-inducible protein 6 | -1 | -4 |
| *PLCE1* | phospholipase C, epsilon 1 | -1 | -3 |
| *DNAH6* | dynein, axonemal, heavy chain 6 | -1 | -5 |
| *SEMA3B* | secreted, (semaphorin) 3B | -1 | -3 |
| *SHISA6* | shisa family member 6 | -1 | -4 |
| *SLFN13* | schlafen family member 13 | -1 | -3 |
| *TPPP3* | tubulin polymerization-promoting protein family member 3 | -1 | -5 |
| *SDK2* | sidekick cell adhesion molecule 2 | -1 | -8 |
| *SMOC2* | SPARC related modular calcium binding 2 | -1 | -2 |
| *LGR5* | leucine-rich repeat containing G protein-coupled receptor 5 | -1 | -4 |
| *ASS1* | argininosuccinate synthase 1 | -1 | -2 |
| *PIFO* | primary cilia formation | -1 | -3 |
| *MUC16* | mucin 16, cell surface associated | -1 | -2 |
| *COL14A1* | collagen, type XIV, alpha 1 | -1 | -3 |
| *DST* | dystonin | -1 | -4 |
| *MS4A8* | membrane-spanning 4-domains, subfamily A, member 8 | -1 | -6 |
