# Supplementary material for: Expression of NAD(P)H quinone dehydrogenase 1 (NQO1) is increased in the endometrium of women with endometrial cancer and women with polycystic ovary syndrome
Source: Clin Endocrinol (Oxf). 2017 Aug 18;87(5):557–65. doi: 10.1111/cen.13436 (PMC5697576; doi:10.1111/cen.13436)
Supplement: Supplementary file 4 [file CEN-87-557-s004.docx]

**Supplemental Table 2: Gene ontology of common differentially expressed genes in PCOS and EC endometrium**

| **Ontology** | **Name** | **Category** | **Genes in Ontology** | **Observed** | **Expected** | **Enrichment** | **q value** |
| --- | --- | --- | --- | --- | --- | --- | --- |
| GO:0006928 | biological process | CC | 1189 | 22 | 5.45 | 4.04 | >0.0001 |
| GO:0004918 | cilium axoneme | CC | 52 | 7 | 0.24 | 29.06 | >0.0001 |
| GO:0005576 | extracellular region | CC | 2140 | 30 | 9.91 | 3.03 | >0.0001 |
| GO:0044441 | cilium part | CC | 110 | 8 | 0.51 | 15.70 | >0.0001 |
| GO:0005930 | axoneme | CC | 70 | 7 | 0.32 | 21.59 | >0.0001 |
| GO:0030286 | dynein complex | CC | 37 | 5 | 0.17 | 29.17 | >0.0001 |
| GO:0005929 | cilium | CC | 239 | 9 | 1.11 | 8.13 | >0.0001 |
| GO:0044421 | extracellular region part | CC | 1099 | 18 | 5.09 | 3.54 | >0.0001 |
| GO:0042995 | cell projection | CC | 1230 | 18 | 5.70 | 3.16 | 0.0002 |
| GO:0005874 | microtubule | CC | 338 | 1.57 | 1.57 | 5.75 | 0.0003 |
| GO:0031012 | extracellular matrix | CC | 426 | 10 | 1.97 | 5.07 | 0.0003 |
| GO:0005578 | proteinaceous extracellular matrix | CC | 360 | 9 | 1.67 | 5.40 | 0.0005 |
| GO:0031838 | haptoglobin-hemoglobin complex | CC | 3 | 2 | 0.01 | 143.91 | 0.0007 |
| GO:0005875 | extracellular space | CC | 856 | 13 | 3.97 | 3.28 | 0.0009 |
| GO:0003777 | microtubule motor activity | MF | 76 | 6 | 0.34 | 17.74 | 0.0001 |
| GO:0031720 | haptoglobin binding | MF | 2 | 2 | 0.01 | 224.76 | 0.0008 |
| GO:0003774 | motor activity | MF | 131 | 6 | 0.58 | 10.29 | 0.0008 |

**CC: cellular component, MF: molecular function**
