# Supplementary material for: Expression of NAD(P)H quinone dehydrogenase 1 (NQO1) is increased in the endometrium of women with endometrial cancer and women with polycystic ovary syndrome
Source: Clin Endocrinol (Oxf). 2017 Aug 18;87(5):557–65. doi: 10.1111/cen.13436 (PMC5697576; doi:10.1111/cen.13436)
Supplement: Supplementary file 5 [file CEN-87-557-s005.docx]

|  |  |  |  |  |
| --- | --- | --- | --- | --- |

**Supplemental Table 3. Univariate analysis of known clinicopathological variables and NQO1 score and their association with survival**

| **Variable** | **Overall survival, HR (95% CI)** | **p value** | **Cancer specific survival, HR (95% CI)** | **p value** | **Recurrence free survival, HR (95%CI)** | **p value** |
| --- | --- | --- | --- | --- | --- | --- |
| Age (1yr) | 1.098(1.049- 1.149) | <0.0001**** | 1.079 (1.017-1.145) | 0.011* | 1.064 (1.025-1.106) | 0.001*** |
| Grade* | 24.124(3.246-179.26) | 0.002** | 69.179 (0.780-6137.205) | 0.064 | 7.484 (2.598-21.557) | 0.0002*** |
| Stageᶧ | 5.279 (2.308-12.078) | <0.0001**** | 8.754 (2.682-28.568) | 0.0003*** | 4.440 (2.132-9.248) | <0.0001**** |
| Histological type | 29.217(3.933-217.035) | 0.001*** | 84.761 (0.911-7885.842) | 0.055 | 7.257 (2.764-19.057) | <0.0001**** |
| LVSI | 2.593 (1.054-6.380) | 0.038* | 3.945 (1.080-14.411) | 0.038* | 3.066 (1.386-6.780) | 0.006** |
| Depth of myometrial invasion | 1.395 (0.610-3.191) | 0.430 | 0.902 (0.301-2.702) | 0.853 | 1.210 (0.584-2.508) | 0.608 |
| NQO1 score | 1.821 (0.786-4.218) | 0.162 | 1.880 (0.631-5.599) | 0.257 | 1.781 (0.859-3.693) | 0.121 |
